# Immunohistochemical consistency between primary tumors and lymph node metastases of gastric neuroendocrine carcinoma

**DOI:** 10.1186/1477-7819-10-115

**Published:** 2012-06-22

**Authors:** Chieko Uchiyama, Shigeyuki Tamura, Shinichi Nakatsuka, Atsushi Takeno, Hirofumi Miki, Takashi Kanemura, Shin Nakahira, Rei Suzuki, Ken Nakata, Yutaka Takeda, Takeshi Kato

**Affiliations:** 1 Department of Surgery, Kansai Rousai Hospital, 3-1-69 Inabaso, Amagasaki City, Hyogo 660-8511, Japan; 2 Department of Pathology, Kansai Rousai Hospital, 3-1-69 Inabaso, Amagasaki City, Hyogo 660-8511, Japan

**Keywords:** Ki67, Immunohistochemistry, Heterogeneity

## Abstract

**Background:**

Gastric neuroendocrine carcinoma (G-NEC) is a rare, highly malignant tumor that exhibits aggressive growth leading to vascular invasion, distant metastasis and extremely poor prognosis. We studied the clinicopathological findings of seven patients at our institute to better under this disease.

**Methods:**

Seven cases of G-NEC were identified among 1,027 cases of gastric carcinoma that underwent gastrectomy at Kansai Rousai Hospital between 2002 and 2010. We studied the pathological and immunohistochemical features of gastric neuroendocrine carcinomas at both the primary site and metastatic lymph nodes.

**Results:**

The mean patient age was 73 years (range 63 to 86 years). There were no females in this series. The final staging was Stage I in one case, Stage II in two, Stage III in two and Stage IV in two. A total of 31 metastatic lymph nodes were found in these patients. This study revealed that the ratio of neuroendocrine cells was similar between the primary and metastatic sites, which tended to show the same expression patterns of neuroendocrine markers.

**Conclusions:**

Metastatic lymph nodes showed heterogeneous immunohistochemical expression patterns similar to the primary sites. G-NEC is far advanced at diagnosis and rapidly reaches the lymph nodes retaining its heterogeneity, carrying a worse prognosis than common gastric cancer.

**Mini abstract:**

G-NEC grows rapidly and metastasizes to the lymph nodes, retaining its pathological and immunohistochemical heterogeneity even at the metastatic sites.

## Background

Gastric neuroendocrine carcinoma (G-NEC) is a rare tumor (0.1 to 0.2% of all gastric carcinomas) with highly malignant biological behavior exhibiting aggressive growth that leads to vascular invasion, distant metastasis and extremely poor prognosis. The 2010 WHO classification defines well-differentiated endocrine tumors/carcinomas as neuroendocrine tumors (NETs), and poorly differentiated endocrine carcinomas as neuroendocrine carcinomas (NECs). Compared with well-differentiated gastric NETs, G-NECs have highly malignant behavior and poor prognosis, but their prognostic markers and therapeutic strategies have not yet been defined.

A definite diagnosis of G-NEC is provided by immunohistochemical examination with neuroendocrine markers, such as synaptophysin (SYN), chromogranin A (CGA), CD56 and neuron-specific enolase (NSE). It has been proposed that care should be exercised in diagnosis because of the variation shown by G-NECs in both histological morphology and immunohistochemical expression. However, no reports have investigated the relationship between the expression pattern at the primary site and that at the metastatic sites as to both histological morphology and immunohistochemical expression. In this study, we examined the primary tumors and all metastatic lymph nodes, and reviewed the association of expression patterns by means of immunohistochemical examination.

## Methods

### Patients and specimens

Seven cases of G-NEC were identified among 1,027 cases of gastric carcinoma that underwent gastrectomy at Kansai Rousai Hospital between 2002 and 2010 (0.68%). All patients gave written informed consent for clinicopathological evaluation.

Table 1 lists the clinicopathological characteristics of these patients. The median age was 73 years (range 63 to 86 years). There were no females in this series. All patients underwent gastrectomy with regional lymph node dissection, and in Case 4, additional liver resection was performed for synchronous liver metastasis.

**Table 1:** Patients’ characteristics

**Patient No.**	**Age**	**Location**	**Gross type**	**Tumor size (cm)**	**Preoperative diagnosis**	**Operation**	**R**
1	63	L	3	13	tub2	Distal gastrectomy	R2
2	71	M	2	2	por1 > tub2	Total gastrectomy	R0
3	71	M	3	13	NEC	Distal gastrectomy	R1
4	86	M	2	3	tub2 > por1 > por2	Distal gastrectomy + Hepatectomy	R1
5	74	M	2	9	NEC	Distal gastrectomy	R0
6	69	L	3	3	tub2	Distal gastrectomy	R0
7	77	M	5	6	NEC	Distal gastrectomy	R0

NEC, neuroendocrine carcinoma; por1, poorly differentiated adenocarcinoma, solid type; por2. poorly differentiated adenocarcinoma, non-solid type; R, Resectability; tub2, moderately differentiated tubular adenocarcinoma.

### Immunohistochemical staining

All resected stomachs and lymph nodes were fixed in 10% neutral formalin, and then, the entire tumor was step-cut to a width of 4 to 5 mm. Specimens were embedded in paraffin, cut into 4-μm sections and stained with hematoxylin and eosin. An immunohistochemical procedure using the EnVision system (DakoCytomation, Glostrup, Denmark) was employed as previously established. Immunohistochemical staining was examined in all blocks of the maximum divided surface of the primary site and all blocks of the metastatic lymph nodes in each case. We reviewed the histology of the tumor on hematoxylin and eosin stains and evaluated the expression of SYN, CGA, CD56 and NSE. We examined not only the primary tumors but also 236 lymph nodes, from which we identified 31 metastatic lymph nodes derived from five patients. The immunohistochemical expression for each antibody was defined as follows: − (<5%), 1+ (5 to 9%), 2+ (10 to 49%) and 3+ (over 50%). The ratio of the tumor area with positivity of each marker was evaluated throughout the maximum dimension of the primary tumors and metastatic lymph nodes. Positivity was defined as a dimension ratio of expression exceeding 10% (over 2+). The following antibodies were used: anti-synaptophysin and anti-CD56 purchased from Novocastra Laboratories Ltd. (Newcastle upon Tyne, UK), and anti-chromogranin A and anti-NSE purchased from DakoCytomation (Glostrup, Denmark). The Ki67 labeling index was also estimated in the block including the deepest part of the primary tumor.

## Results

### Gross findings and staging

Five tumors were located in the middle of the stomach, and the remaining two were in the lower stomach (Table 1). One case was a T1b (submucosa) tumor, four cases were T3 (subserosa), one was T4a (penetration of serosa) and one was T4b (invasion to adjacent structures) (Table 2). Lymph node metastasis was found in five cases. The final staging was Stage IA in one case, Stage IIA in one, Stage IIB in one, Stage IIIA in one, Stage IIIB in one and Stage IV in two, according to the seventh American Joint Committee on Cancer (AJCC) TNM staging classification. The tumors had grown to a median size of 6 cm (range 2 to 13 cm) in the greatest dimension. Grossly, the tumor was Type 3 in three cases, Type 2 in three and Type 5 in one.

**Table 2:** Clinicopathological findings of primary tumor and resected lymph node

**Pt No**	**Pathological Stage**							**Metastatic ratio of dissected lymph nodes**		**Histology of lymph node metastases**					
										**Ad**		**Concomitant Ad and NEC**		**NEC**	
								**n**	**(%)**	**n**	**(%)**	**n**	**(%)**	**n**	**(%)**
1	T4b	N3a	M0	H0	P0	CY1	IV	8/16	(50)	0	(0)	0	(0)	8	(100)
2	T3	N2	M0	H0	P0	CY0	IIIA	5/26	(19)	0	(0)	0	(0)	5	(100)
3	T3	N0	M0	H0	P0	CY0	IIA	0/42	(0)			-		-	
4	T3	N1	M0	H1	P0	CY0	IV	1/31	(3)	0	(0)	0	(0)	1	(100)
5	T3	N1	M0	H0	P0	CY0	IIB	2/52	(4)	0	(0)	0	(0)	2	(100)
6	T4a	N3b	M0	H0	P0	CY0	IIIC	15/25	(60)	12	(80)	3	(20)	0	(0)
7	T1b	N0	M0	H0	P0	CY0	IA	0/44	(0)	-		-		-	

### Expression of immunohistochemical staining between the primary and metastatic sites

**Figure 1:**
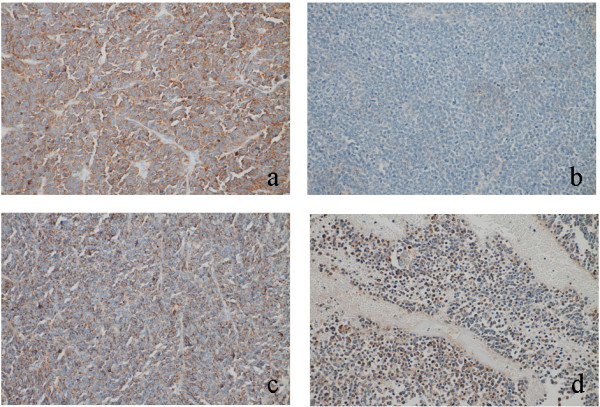
**Immunohistochemical expression for neuroendocrine markers in primary tumor (Case 1). a**) Synaptophysin, **b**) chromogranin A, **c**) CD56, **d**) neuron-specific enolase. Tumor cells variably expressed neuroendocrine markers.

**Figure 2:**
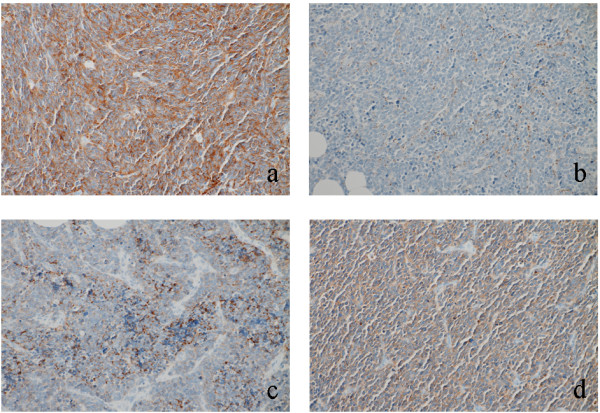
**Immunohistochemical expression for neuroendocrine markers in metastatic tumor cells in lymph nodes (Case 1). a**) Synaptophysin, **b**) chromogranin A, **c**) CD56, **d**) neuron-specific enolase. Expression patterns of neuroendocrine markers in metastatic tumors were similar to those in the primary tumor.

Only three cases were correctly diagnosed as G-NEC preoperatively. We summarized the varied histological patterns of the primary tumors and lymph nodes in Table 3. According to the WHO classification, five of the seven tumors were large-cell subtypes and the others were small-cell subtypes. They were classified into five pure neuroendocrine carcinomas and two tumors combined with adenocarcinoma. We examined a total of 236 lymph nodes obtained from seven patients. Among them, 31 positive nodes included 12 nodes showing pure adenocarcinoma, 3 showing both adenocarcinoma and neuroendocrine carcinoma, and 16 showing pure neuroendocrine carcinoma. While the cases with pure NEC had lymph node metastasis of pure NEC (for example, Case 1 shown in Figures 1 and 2), the two remaining cases with both adenocarcinoma cells and neuroendocrine carcinoma cells had varied lymph node metastasis. Case 4 had pure NEC, and Case 6 had both pure adenocarcinoma nodes and concomitant nodes. In addition, we verified the primary and lymph node subtypes by staining for neuroendocrine markers, and all primary tumors were strongly stained by SYN and CD56. Most cases showed varied expression patterns that were similar in both the primary and metastatic sites. The accordance of positivity between the primary sites and lymph nodes was extremely high: 80% in SYN, 100% in CGA, 60% in CD56 and 80% in NSE. Furthermore, the Ki67 labeling index was high, over 20% in all cases.

**Table 3:** Histology and immunohistochemical findings of both primary tumor and metastatic lymph nodes

**Patient no.**	**Primary tumor**						**Lymph node metastases**					**Ki-6 7 labeling index (%)**	**Mitotic counts (/10HPF)**
	**Histology**	**Ratio of neuroendocrine cell (%)**	**Expression of neuroendocrine markers**				**Histlogy**	**Expression of neuroendocrine markers**					
			**SYN**	**CGA**	**CD56**	**NSE**		**SYN**	**CGA**	**CD56**	**NSE**		
1	SC	100	3+	-	3+	3+	SC	3+	-	3+	3+	60	60 to 70
2	LC	100	3+	3+	2+	1+	LC	1+	3+	1+	2+	20	20
3	LC	100	3+	-	2+	3+						80	80 to 90
4	LC > tub2	90	3+	3+	3+	-	LC	3+	3+	2+	-	30	80 to 90
5	LC	100	3+	-	3+	-	LC	3+	-	3+	-	70	100 to 110
6	LC > tub2 > por1	60	3+	3+	3+	1+	pure Ad	2+	-	-	-	70	20
							LC + Ad	3+	2+	1+	-		
7	SC	100	3+	-	2+	3+						80	100 to 110

Expression positivity was defined as follows; - (<5%), 1+ (5 to 9%), 2+ (10 to 49%), and 3+ (over 50%). Ad, adenocarcinoma; CGA, chromogranin A; HPF, high power fields; LC, large cell; NSE, neuron-specific enolase; SC, small cell; SYN, synaptophysin.

### Clinical course

Adjuvant chemotherapy, including S-1, was introduced to all cases but one at an early stage. The median treatment duration was 8.1 months (range 0.9 to 24.5 months). During the treatment course, there were two recurrences (Cases 1 and 4) and one death three months after incurative surgery (Case 1). The major site of relapse was the liver, followed by the peritoneum. Chemotherapy was introduced after liver recurrence in Case 4. The patient responded well to the therapy and achieved long, overall survival, 21 months, despite his advanced stage. The three-year disease-free survival rate was 64.3%, and the three-year overall survival rate was 83.8% after surgery.

## Discussion

NECs are classified into pure tumors and composite tumors admixing adenocarcinomatous differentiation [1,2]. Criteria for classification in NEC categories is that over 30% of the cells display the features of neuroendocrine differentiation [2]. NECs have aggressive biological behavior and exhibit rapid proliferation [1-7].

We studied the characteristics of G-NECs by means of pathological and immunohistochemical examination of both the primary sites and metastatic lymph nodes. In this study, we found an admixed population of pure neuroendocrine cells, adenocarcinoma cells and their intermediate cells that have the morphological features of adenocarcinoma with positivity to neuroendocrine markers. Other studies have reported that NEC shows strong staining for neuroendocrine markers, such as CGA, SYN, NSE and CD56 [1-3,5,7]. In our series, the positivity rate for CGA, SYN, NSE and CD56 was 42.9%, 100%, 85.7% and 72.7%, respectively. Among these markers, tumors showed the highest positivity for SYN and lowest positivity for CGA. CGA is a marker for neuroendocrine granules and an indicative factor of differentiation to neuroendocrine cells. The poor differentiation in our cases prevented sufficient expression of granules.

We found that the tumors varied in immunohistochemical expression. In one tumor, while some cells with high CGA expression showed negativity for other markers, some cells with no CGA expression showed diffuse high positivity for other markers. In addition, besides neuroendocrine cells positive for SYN, some adenocarcinoma cells showed positivity for SYN among composite-type tumors. This indicates histological and immunophenotypical continuity between the adenocarcinoma component and the NEC component.

Furthermore, focusing on lymph nodes, we found that cases with pure neuroendocrine primary sites had metastatic lymph nodes with pure neuroendocrine cells (Table 3). The variation in immunohistochemical expression patterns of the primary site was maintained even in the metastatic pure endocrine cells. In Case 6, one of the two cases with combined primary tumors, composite metastasis of NEC and adenocarcinoma was seen in some lymph nodes, and pure adenocarcinoma metastasis was seen in other nodes. The tumor showed consistency in both histological type and immunohistochemical expression between the primary site and the metastatic lymph nodes. This result led to a hypothesis as to the manner of metastasis. That is, clustered cells, including adenocarcinoma and neuroendocrine cells with varied immunophenotypes, spread to the lymph nodes and coexisted there. Additionally, lymph nodes of composite-type tumors expressed a slightly different staining pattern. Cells not undergoing sufficiently mature differentiation in primary sites may differentiate into neuroendocrine cells or develop varied immunohistochemical expression. In this series, there was no clear association between immunohistochemical expression and clinical outcome. Cases with low Ki67 labeling indices had a good prognosis. For example, Case 2, for which the Ki67 labeling index was 20%, achieved the longest survival term, 55 months. Case 4, for which the Ki67 labeling index was 30%, was successfully treated with surgery and chemotherapy and survived for 21 months despite distant metastasis. Limitations of this study include short duration of follow-up and small sample size mostly composed of pure types. Therefore, large-scale and long-term studies are needed to draw a definitive conclusion.

## Conclusion

In summary, we reported the pathological and immunohistochemical features of neuroendocrine carcinomas at both the primary sites and metastatic lymph nodes. The cells grow rapidly and metastasize to the lymph nodes retaining their heterogeneity even at the metastatic sites.

## Abbreviations

G-NEC: Gastric neuroendocrine carcinoma; NETs: neuroendocrine tumors; NECs: neuroendocrine carcinomas; SYN: synaptophysin; CGA: chromogranin A; NSE: neuron-specific enolase; por1: poorly differentiated adenocarcinoma, solid type; por2: poorly differentiated adenocarcinoma, non-solid type; R: Resectability; tub2: moderately differentiated tubular adenocarcinoma; AJCC: American Joint Committee on Cancer; Ad: adenocarcinoma; HPF: high power fields; LC: large cell; SC: small cell.

## Competing interests

The authors declare that they have no competing interests.

## Authors’ contributions

CU and ST conceived of the study. ST and SN supervised the manuscript writing. SN performed the pathological and immunohistochemical evaluation and scoring. AT, HM, TK, SN, RS, KN, YT and TK collected the cases and clinical information. CU performed the literature review and wrote the manuscript. AT and SN performed the statistical analysis. All authors read and approved the final manuscript.
